# On the Contagiousness or Non-Contagiousness of Yellow Fever

**Published:** 1805-11-01

**Authors:** Thomas Dancer

**Affiliations:** Kingston, Jamaica


### Medical and Phyſical Journal.

VOL. XIV.] NOVEMBER 1, 1805. [NO. 82.

### ON THE CONTAGIOUSNESS OR NON-CONTAGIOUSNESS OF YELLOW FEVER; BY THOMAS DANCER, M. D OF *Kingston, Jamaica.*

( Communicated by Dr. ROLLO. )

IT is greatly to be lamented that a due reverence for long established doctrines on the one hand, and a laudable ardour for discovery on the other, should alike tend to mislead us in onr researches, and that the investigation of truth, in a matter of the greatest moment to mankind; one on which the very existence of society depends, should be attended with a difficulty almost insuperable. How else could it happen that so many medical men, distinguished not only for their talents, but for a spirit of liberal and candid enquiry, should differ so widely from each other, and at a short distance of time, *from themselves*; many of them having been led to change their opinion on the subject: which shews that plain and simple as it may by some be conceived, it is in reality not so, but involved in much intricacy. I will not presume that what I have to offer on the subject will be considered as completely satisfactory; but if I can bring medical men, who are disposed to view it without prejudice, and apart from all other matters with which it is connected, (viz. quarantine, &c.) to a nearer point of agreement, my endeavours will not be fruitless.

" *When* (as Dr. Seaman observes), *facts are more scrutinized,* the advocates *for either opinion will possibly find some ground for compromise.*" This is an event devoutly to be wished, for the differences that at present subsist do not redound to the good of mankind, nor to the credit of the profession.

A science like medicine does not always admit of definite language; this is apparent on many occasions, and is partly so, if not particularly so, with respect to the terms, Infection and Contagion, from the *confounding* of which, or from assigning to each, *new and* not *well*

*defined significations*, proceeds in a great measure the obscurity that rests on the question under discussion. Their etymological import, so critically ascertained in a late ingenious Thesis, by Dr. Bayley, is of little moment. It is the sense in which they are ordinarily accepted or understood,* by which we are to be guided in this enquiry; and adhering to this, the pestilential yellow fever† must be considered as frequently contagious, that is to say, propagated by emanations proceeding from the bodies of those labouring under it. But if we are to adopt the new coined distinction between infections and contagions, viz. that contagious diseases are such only as arise from a specific matter secreted, and, like the small-pox, &c. affect the constitution but once during life, then there is an end to the dispute. But is this novel distinction so obvious as to be understood, and so satisfactory as to be admitted? ‡ Do none of the diseases depending on a specific virus affect the constitution but once? Does it appear that there is no difference between *animal* and *vegetable* putrefaction? or can the emanations from the living diseased body be considered as of the same nature with those from dead animal matter? [See Ferriar on Contagion; — there are few instances of diseases caught in dissecting rooms.]

May

---

* Fevers of infection have hitherto (at least since the time of Sir John Pringle) been considered as arising from marsh miasmata and putrid exhalations; while *contagious* fevers were supposed to be occasioned by emanations or effluvia from sick bodies and excrementitious matters.

† Some ambiguities on the subject may arise from confounding the endemial yellow fever with the malignant or pestilential yellow fever. The diagnosis between them, on some occasions, may be difficult, but generally they may be distinguished.

1st, In the malignant yellow fever there is seldom more than one paroxysm, continuing for a longer or shorter period, and terminating in death or recovery.

2d. In the pestilential yellow fever all the symptoms run much higher, and there are many marks of malignancy that are peculiar, viz. sphacelus of the scrotum.

‡ The advocates for this new distinction, between infectious and contagious diseases, are not agreed in this mode of classing diseases. By one, *Lues Venerea* (because depending on a specific virus) is denominated contagious. It would be well for mankind if it answered to the definition, and occurred only once to the same person. Rather than allow yellow fever to be contagious, like jail, hospital fever, &c. it is denied that the latter are so. Dr. Cathral says, these do not depend on a putrefactive process but on vitiated secretion; and why may not yellow fever depend on the same

May not the effluvia of the human body under disease, be supposed to be of a very different nature from what they are in health, and *specifically* so, under many diseases, as in influenza, &c.

We are confessedly alike ignorant with respect to the matter of infection and contagion;* but is there any just ground to conclude that all fevers arise solely from one and the same cause? That jail fever, hospital fever, pestilential yellow fever, originate in the same way as intermittents and remittents, which evidently depend on causes existing in the atmosphere?† If this were the case, why should not the phenomena, under each, be the same, but they are not so; we see an essential difference amongst them; they do not exist in the same seasons and situations; they do not run the same course, nor have they the same terminations.

It has been supposed, that if animal contagion and miasmata or putrid exhalations are not specifically the same, they have an intimate connection; but there is no necessity for any such supposition; they only serve reciprocally to dispose the body to be acted on like other occasional causes.‡

That the noxious effluvia arising from diseased bodies may adhere to clothes, bedding, &c. can scarcely be called

 in same? and how do vitiated secretions act but by the effluvia arising from them?

* It is indeed contended, that one consists of *hydrogen* or *sulphurated hydrogen gas*, and the other on the gaseous oxyd of septon. Far be it from me to throw any contempt on the new discoveries in chemistry; but I cannot be of opinion that we have as yet made sufficient progress in the science to be able to explain the composition of pestilential effluvia, no more than of the specific contagion of small-pox, measles, poison, &c. Are not all these, as well as odours, combinations of azote, hydrogen, oxygen, and carbon? But what explanation does this afford? Who can say in what proportion, or shew the quo modo of their operation?

† I am sorry to say that there is a fashion in philosophy as in every thing else. It is the ton at present to talk of the *grades* of fever, and of its different *states*, or the gangrenous state, &c. as though all fevers were essentially the same, originating in the same causes, running the same course, and having the same tendency and termination. I am a disciple of the Old School, but no bigot; I am sensible of the imperfections in the present state of medicine, but I cannot allow myself to be carried away by every novelty.

‡ Dr. Rush mentions a very singular circumstance. If a person (says he) who had taken the yellow fever in Philadelphia, afterwards went into a family, in question, there are so many histories in direct proof. Some of the non-contagionists do not refuse to allow that the cause *may be so imported,* but they nevertheless insist that it cannot by such means be communicated and become epidemic. Is not this assertion without proof, or rather against established facts and experience.

It appears to me, therefore, that however sufficient local causes may be to *generate* a fever * of this kind, though all the arguments so industriously collected, and so strenuously urged against the occasional contagiousness of the yellow fever, are far from being conclusive. Yellow fever, like jail, ship, and hospital fever, being once generated, is susceptible of being propagated in the same way, viz. by effluvia proceeding directly from sick bodies, or else adhering to clothes, &c. &c.; and according to the observation of an intelligent writer on the subject, *"it is not improbable that the spark which first kindled up the epidemic might have been imported."*†

I shall now state the reasons and facts on which I ground my opinion, as to the contagious nature of yellow fever.

1st. I refer to the numerous facts recorded by Dr. Chisholm, which though called in question, have not, in my opinion, been invalidated.

2d. These facts have been confirmed by various other persons, both in America and the West Indies. [See Med. Reports.]

3d.

---

mily, living near the river, or in an unhealthy situation, the same disease appeared in several instances in the family. This, by persons of common understanding, might be deemed proof positive of its contagious nature; but no! Dr. R. is a non-contagionist, and therefore denies it. How then does he explain the paradox? Why, says he, the exhalations arising from persons under the yellow fever, act as a *stimulus,* and dispose the body to be acted on by other causes. This is what the Contagionists will allow, but then they will insist that this stimulus is a *specific one,* or else it could not produce the yellow fever. If not specific, it might as well produce any other disease.

* There is no contradiction in saying, that a disease may be *generated* and *contagious.* How did the contagion, small-pox, &c. first originate? No one will doubt of the contagion of hydryphobia, and yet that is at first sporadic or affecting an individual.

† Some persons, rather than allow of the existence of contagion in any case whatever, refer epidemic diseases to unknown constitutions of the atmosphere, or occult qualities in it, to a decomposition in it by caloric, to animalcula, earthquakes, volcanos, &c. The learned and famed Dr. Moseley has made the rare discovery, that the plague is not contagious; and that the *buboes* in plague, are owing to the gross feeding of the Turks. How kind is Providence to the English nation! that they have not buboes from coarse feeding.

3d. It is well known to the practitioners in this island, that the pestilential yellow fever prevails only on certain occasions; that it generally begins among sailors, soldiers, and others newly arrived from Europe; that it has no connection with local causes; that is to say, it makes its appearance indiscriminately, in all seasons and situations, or rather it may be said to prevail most in situations remote from marshes and swamps, which generate bilious remittents, not yellow fever.

It appears also (contrary to what has been again and again affirmed) to be communicable from one person to another, and from a single person to many; *e. g.* one man only going on board an infected ship, and contracting the disease, has communicated it to several others who had been no otherwise exposed to it.

Sailors walking from one post to another, and being taken ill at plantations, when they have called in for lodging and refreshment, have communicated the yellow fever to the Book-keepers and Overseers. A late instance occurred, when no less than three persons were carried off by infection so received. Many other histories might be adduced, in proof of what I have just now stated. I have information from several country practitioners, that at the out posts, the crews of ships after their arrival are generally healthy, till one or more are taken sick; but that as soon as a single instance of Yellow Fever occurs, the infection spreads from ship to ship, through the whole.

The disease has also been traced from plantation to plantation, 15 miles from the shipping, into the interior of the country. But the strongest evidence of the contagious nature of yellow fever I have yet met with, I have been favoured with by Dr. D. Brown, of the Royal Artillery, formerly Assistant Surgeon to the 6th Battallion of the 60th Regiment, quartered at Up Park Camp.

*Copy of* Dr. Brown's *Letter, to* Dr. Dancer, *dated Military Hospital, Up Park Camp, September* 10, 1803.

Dear Sir,

In respect to the fever that has lately raged with such violence in our hospital; it was without doubt contagious. The reasons I have for being of that opinion, are shortly these.

1st. No local cause existed, that could produce the disease.

2dly. It went progressively through the battalion, from the Easternmost or windward point, the persons nearest to the sick being always the first that were attacked.*

3dly. The medical gentlemen were all taken ill; one died, and the rest recovered with difficulty.

4thly. The sergeants and orderly men were all affected, and many of them died. But,

5thly. What was most singular, every man employed in shaving the sick, fell a victim.

These circumstances are known to every officer and private in the Regiment.

I am, &c.

DAVID BROWN.

Any comment on the above mentioned facts must be superfluous; but I cannot conclude this paper without expressing my surprise and regret, at the zeal shewn by several late writers, in their attempts to subvert uncontrovertible facts, with respect to the real existence of contagion. To deny that the *Plague* is contagious, is to be guilty of a libel on the common sense and veracity of mankind, and to shake the foundation of all human testimony; but see what credit strong assertion will gain! The desire of being distinguished for superior and original thinking, what will it not tempt men to, who court not the truth, but public fame?

In this extraordinary age, a man must affect a supreme contempt for every thing belonging to former times.

Our sons they count us fools, so wise they grow;
No doubt, their wiser sons will think them so.

* The situation of Up Park Camp is as healthy a one as can be well imagined. It is considerably elevated above the sea, and distant from it only two miles, so as to be fully exposed to the sea breezes. There are no swamps nor sources of putrefaction in the neighbourhood. The barracks not crowded, nor filthy. The hospital may be considered a model for all others, &c Yet, notwithstanding all these circumstances, so favourable to the health of the soldiers, upwards of 90 men died in the space of a few weeks, with a fever, characterized with all the symptoms of yellow fever; and this happened during the healthy season of the year, June and July.

CASE

